# A Survey on the Use and Barriers of Surface Electromyography in Neurorehabilitation

**DOI:** 10.3389/fneur.2020.573616

**Published:** 2020-10-02

**Authors:** Andrea Manca, Andrea Cereatti, Lynn Bar-On, Alberto Botter, Ugo Della Croce, Marco Knaflitz, Nicola A. Maffiuletti, Davide Mazzoli, Andrea Merlo, Silvestro Roatta, Andrea Turolla, Franca Deriu

**Affiliations:** ^1^Department of Biomedical Sciences, University of Sassari, Sassari, Italy; ^2^Department of Rehabilitation Sciences, KU Leuven, Leuven, Belgium; ^3^Department of Rehabilitation Medicine, Amsterdam UMC, Amsterdam Movement Sciences, Amsterdam, Netherlands; ^4^Department of Electronics and Telecommunications, Politecnico di Torino, Turin, Italy; ^5^Human Performance Laboratory, Schulthess Clinic, Zurich, Switzerland; ^6^Gait and Motion Analysis Laboratory, Sol et Salus Hospital, Rimini, Italy; ^7^Department of Neuroscience, University of Torino, Turin, Italy; ^8^Laboratory of Rehabilitation Technologies, Istituto di Ricovero e Cura a Carattere Scientifico (IRCCS) San Camillo Hospital, Venice, Italy

**Keywords:** surface electromyography, sEMG, neurorehabilitation, survey, expert opinion, muscle activation, clinical research

## Abstract

Historical, educational, and technical barriers have been reported to limit the use of surface electromyography (sEMG) in clinical neurorehabilitation settings. In an attempt to identify, review, rank, and interpret potential factors that may play a role in this scenario, we gathered information on (1) current use of sEMG and its clinical potential; (2) professional figures primarily dealing with sEMG; (3) educational aspects, and (4) possible barriers and reasons for its apparently limited use in neurorehabilitation. To this aim, an online 30-question survey was sent to 52 experts on sEMG from diverse standpoints, backgrounds, and countries. Participants were asked to respond to each question on a 5-point Likert scale or by ranking items. A cut-off of 75% agreement was chosen as the consensus threshold. Thirty-five invitees (67%) completed the electronic survey. Consensus was reached for 77% of the proposed questions encompassing current trends in sEMG use in neurorehabilitation, educational, technical, and methodological features as well as its translational utility for clinicians and patients. Data evidenced the clinical utility of sEMG for patient assessment, to define the intervention plan, and to complement/optimize other methods used to quantify muscle and physical function. The aggregate opinion of the interviewed experts confirmed that sEMG is more frequently employed in technical/methodological than clinical research. Moreover, the slow dissemination of research findings and the lack of education on sEMG seem to prevent prompt transfer into practice. The findings of the present survey may contribute to the ongoing debate on the appropriateness and value of sEMG for neurorehabilitation professionals and its potential translation into clinical settings.

## Introduction

Surface electromyography (sEMG) is a technique for non-invasive measurement of the electrical activity of a muscle through adequately positioned surface electrodes on the skin (1). sEMG has been suggested as a tool to enhance neuromuscular assessment and rehabilitation of individuals with neurologic conditions. Although sEMG has been used extensively for research and its potential value in neurorehabilitation has been proposed (2–4), the true benefits that this technology may bring to clinicians and patients are unclear, possibly limiting its translational use in clinical practice. Based on a recent qualitative study conducted among neurorehabilitative experienced personnel (5), sEMG was deemed by clinicians as hardly compatible with practical aspects of rehabilitation, with limited time and resources perceived as the most relevant barriers to its employment. Transferring medical innovations into clinical practice is quite difficult in rehabilitation (6, 7). Two main reasons have been identified for this challenging translation: (1) limited knowledge of the relevant physical laws and conditions that apply to problems or circumstances, in contrast to evidence-based practice (EBP) guidelines and recommendations; (2) failure of an individual or group to apply established knowledge correctly in a specific circumstance (7, 8). According to Jette (7), these limitations may very well apply to the rehabilitation domain. Indeed, while EBP provides clinicians with a systematic approach to appraise, select, apply, and integrate research findings with patient preferences and clinicians' expertise as part of their clinical decision-making process, inappropriate, or insufficient adherence to indications results in a limited impact on patients (9). We believe that such line of thought may be stretched to the paradigmatic case of sEMG usage in clinical practice, as the benefits of this technology may not be perceived as compelling enough to support its incorporation into clinical practice, given the current lack of translational evidence.

Due to its non-invasive nature, sEMG has been used as a clinical tool in several neurological conditions such as Parkinson's disease (10, 11), stroke, and cerebral palsy (12), but also in orthopedic and gynecological rehabilitation, in sport, aging, and space medicine, as well as in gnathology (13). Specific examples of routine clinical use include measuring muscle fiber conduction velocity after electrical stimulation of peripheral nerves (14, 15) and as a standard for recording compound muscle action potentials after transcranial or peripheral magnetic stimulation (16). Moreover, integration of sEMG into gait analysis has greatly advanced our understanding of muscle function during typical and pathological gait (17). Other clinical applications in human movement analysis include recording a muscles' reaction time or the synergistic properties of multiple muscles. Recent findings have also shown that sEMG can be useful for neurorehabilitation (3–5) in the context of physician-supervised programs designed to rehabilitate people with diseases, traumas, or disorders of the nervous system. Ample evidence exists for its use in predicting long-term recovery from neurologic injury such as stroke and spinal cord injury, understanding healthy and pathological muscle activity profiles and interlimb coordination, quantifying dynamic motor control parameters in gait, supporting the design of neuro-orthopedic surgery, providing biofeedback, and tracking the effects of conservative rehabilitation and surgery (18–22). Moreover, methodological recommendations are nowadays available thanks to *ad-hoc* European actions and to the efforts made by national scientific societies (17, 23).

However, since its very first applications to assess human gait in the 1950s and the massive developments of this technology in the 1970s (24), its value as a clinical tool is still controversial (3, 5, 11). In fact, even when integrated into clinical movement analysis, the lack of an accepted gold standard prevents it from being widely considered as an essential component. Indeed, sEMG was generally regarded as an auxiliary tool rather than the main investigation method in the functional evaluation of a patient (16).

In a recent qualitative study, Feldner et al. (5) set out to examine the clinicians' perceived value, benefits, drawbacks, and ideas for technology development and implementation of sEMG recordings in neurologic rehabilitation practice. Semi-structured interviews and focus groups were organized among 22 clinicians in the United States with a rehabilitative background (59% occupational therapists, 32% physiotherapists, 9% physiatrists) from inpatient, outpatient, and research settings. The main conclusion of this study was that, despite the acknowledged clinical benefits for neurorehabilitation, sEMG is not routinely employed for assessment or intervention following neurologic injury. Furthermore, limited time and resources were identified as the key barriers to sEMG usage by clinicians. This indicates the need to streamline intuitive and clinically impactful sEMG applications and systems, and to conduct further research to determine the clinical relevance of sEMG in neurorehabilitation, its clinical feasibility and current barriers preventing widespread usage.

In this study, we further expanded the work of Feldner et al. (5) by gathering information from a multidisciplinary panel of experts with different backgrounds to originate experts' opinion on the current use of sEMG and its clinical potential in neurorehabilitation. To achieve this, we departed from Feldner's choice to enroll only clinicians and opted for conducting survey research [which is defined as “the collection of information from a sample of individuals through their responses to questions” (25)] among leading experts who published on sEMG and neurorehabilitation from both a methodological and clinical point of view. Indeed, the present work was conceived and planned as a first step in the establishment of an international, clinical research initiative aimed at appraising the translational value of sEMG in the clinical setting.

Within this framework, we developed questions to gather experts' opinion on: (1) current use of sEMG and its clinical potential in neurorehabilitation; (2) professional figures primarily dealing with sEMG; (3) educational aspects, and (4) possible barriers and reasons for the apparently limited employment of sEMG.

As such, we aimed to outline a common framework and a stimulus for future research on specific sEMG-related issues.

## Methods

An online survey involving experts on sEMG from diverse standpoints, backgrounds (i.e., biomedical engineers, neurophysiologists, kinesiologists, neurologists, physiotherapists) and geographical origins was conducted.

### Participants

Through a literature scan of three biomedical databases (PubMed/Medline, Scopus, Web of Science, as of August 31, 2019) using common keywords (surface electromyography AND neurorehabilitation) and limiting to medical subject headings (MeSH) and MeSH Major Topic, we retrieved an initial set of 821 articles (396 with PubMed/Medline, 247 with Scopus, 178 with Web of Science) to be then manually screened. Based on the title, abstract and keywords, pertinent articles were selected by two of the authors (AM, FD) (87 with PubMed/Medline, 72 with Scopus, 54 with Web of Science). After removing duplicates across the databases, manual screening of the full-texts led to retain 32 articles specifically dealing with the topic under study. From these, a set of unique authors' names was extracted, leaving 82 authors who had published at least two articles which major topic was the application of sEMG to neurorehabilitation from a methodological/technical or clinical perspective, or both. Of these 82 individuals, 52 had authored at least two articles in a prominent role (first or second or last or corresponding author). After extracting contact information, electronic invitations were sent to these 52 authors. The participants were requested to respond anonymously to a questionnaire, which had been developed iteratively by a primary research team (AM, AC, FD) and then reviewed and pilot-tested by an external board of eight “core experts” (LB, AB, UDC, MK, NM, DM, AM, SR, AT). Feedback received during review and piloting was incorporated into the survey.

### Survey Questions

The survey (Supplementary File 1) comprised 30 questions. The last 8 questions (questions 23–30) concerned demographic, background, and professional information, while the 22 remaining questions covered four main themes that had been conceived, shared, and developed into their final form through an iterative process between the primary research team and the external board of core experts. Based on extensive discussion and on experts' suggestions and feedback, the thematic framework was approved as follows: (1) current EMG employment in clinical settings and potential utility (questions 1–10); (2) professional figures using EMG and potential advantages of better qualified professionals (questions 11–15); (3) education, training, and teaching (questions 16–20); (4) potential barriers to sEMG usage (questions 21 and 22).

### The Survey Process

An online software [SurveyMonkey http://survey-monkey.com] was used to deliver the questionnaire electronically. Identified experts were invited to participate via an e-mail that included key information about the study, its purpose, how it would inform consensus on sEMG employment, potential utility, professional figures involved in its usage, education, training and teaching, and potential barriers.

Participants were asked to respond to each question on a 5-point Likert scale (e.g., 1, strongly disagree; 2, disagree; 3, neutral; 4, agree; 5, strongly agree), and in two questions (11 and 21), by ranking items. They were also instructed to leave unanswered those questions that were perceived as outside their expertise/knowledge. We asked them for any additional comments/insights they wished to provide using free-text boxes. These comments were recorded and, based on an eventual trend (i.e., two or more participants raising the same issue), taken into consideration and commented upon when interpreting the survey results. The survey was available for 6 weeks. Four reminders were sent by e-mail to participants on days 14, 28, 35, and 42.

### Data Analysis

A cut-off of 75% agreement was chosen as the consensus threshold based on the findings of a systematic review of surveys and consensus studies (26). Accordingly, we considered consensus to be reached if at least 75% of respondents scored the question 4 or 5 (positive consensus toward agreement) or 1 or 2 (negative consensus toward disagreement) on the 5-point Likert scale. For ranking questions, we analyzed the distribution of the response frequencies and considered only the first three in rank, based on the number of preferences received. Descriptive statistics are reported in the form of counts/proportions/percentages. Subgroup analyses were also carried out to compare the response rates of non-clinical vs. clinical professional figures. The frequency rates were compared using two-tailed Chi-square tests with the significance level set at *p* < 0.05.

## Results

### Participation by Round

Of the 52 invitation e-mails sent (February 24 to April 7, 2020), 35 invitees (67%) completed the 30-question survey. Responses were received from a minimum of 26 to a maximum of 35 participants (74–100%). Data were analyzed from the 35 respondents and the consensus threshold (75%) was calculated for each question relative to the number of respondents.

Table 1 details the respondents' characteristics. Professional background was varied, with 13 (37%) physiotherapists, 12 (34%) biomedical engineers with a focus on instrumentation, e-health, and rehabilitation (hereafter referred to as biomedical engineers), 5 (14%) Physical Medicine and Rehabilitation (PM&R) specialists, also known as physiatrists, 3 (9%) kinesiologists/human movement scientists, and 2 (6%) clinical neurophysiologists. Clinicians accounted for 57% (20/35) of the cohort, of which 17 (85%) were active in the neurorehabilitation field. The other professionals were either engaged purely in research or as lab technicians/engineers in clinical settings.

**Table 1:** Respondents' characteristics (*n* = 35).

**Background**	***n***	**(%)**
Biomedical engineers^*^	12	34
Clinical neurophysiologists	2	6
Kinesiologists/human motion scientists	3	9
PM&R specialists	5	14
Physiotherapists	13	37
**Neurorehabilitation subfield^#^**		
Activities to improve mobility, muscle control, gait, and balance	23	66
Exercise programs to prevent or decrease weakness, manage spasticity and pain, and maintain range of motion	20	57
Help with obtaining assistive devices that promote independence'	12	34
Help with activities of daily living	9	26
Patient's education and counseling	6	17
Not working in a clinical setting	8	23
**Geographical location**		
Australia	1	3
Austria	1	3
Belgium	2	6
Canada	1	3
France	3	9
Germany	1	3
Ireland	1	3
Italy	19	54
Netherlands	2	6
Switzerland	2	6
United Kingdom	1	3
United States	1	3

* *With a focus on instrumentation, e-health, and rehabilitation; PM&R, Physical Medicine and Rehabilitation*;

# *Percent values do not add up to 100 since some respondents identified themselves as part of more than one category*.

The specific clinical subfields of the interviewed cohort were also checked (question 30). The majority of the respondents declared to engage in “Activities to improve mobility (movement), muscle control, gait, and balance” (23/35, 66%) and in “Exercise programs to improve movement, prevent, or decrease weakness caused by lack of use, manage spasticity and pain, and maintain range of motion” (20/35, 57%). Twelve (34%) specifically engaged in “Help with obtaining assistive devices that promote independence,” 9 (26%) in “Help with activities of daily living (ADLs),” and 6 (17%) in “Patient's education and counseling.” Two respondents declared in the comment areas to specifically engage in “Measuring human motion in clinics.” Eight out of 35 (23%) declared not to work in a clinical setting, but rather in clinical research.

Table 2 summarizes all items which exceeded the predefined 75% threshold for consensus. The items for which no consensus was reached are detailed in Table 3.

**Table 2:** Survey items that reached consensus.

**Survey items**	**Agreed**	**(%)**
1. sEMG is more frequently employed in technical/methodological research than clinical research.	29/35	(83)
2. sEMG provides information on neuromuscular function that is not provided by other assessment techniques/tools in neurorehabilitation.	32/35	(91)
3. Practical utility of sEMG in clinical neurorehabilitation. sEMG information on neuromuscular activation may:		
*Enhance the assessment and characterization of neuromuscular impairments in patients*	32/34	(94)
*Influence the intervention plan design*	28/33	(85)
*Allow to better track the changes in muscle activity from baseline when neurorehabilitation interventions are administered*	32/34	(94)
*Allow to evaluate the effects of non-invasive interventions designed to impact muscle activity (such as therapeutic exercise, orthotics, medication, physical agents, manual therapy techniques)*	32/25	(91)
*Allow to evaluate the effects of invasive interventions designed to impact muscle activity (such as surgery and neuromuscular blocks)*	30/34	(88)
*Be employed as biofeedback training if the clinician identifies abnormal patterns of muscle activity that may be modified through motor learning*	30/34	(88)
4. Role of sEMG in patient's assessment—sEMG may be useful to:		
*Outline the sequential timing of muscular actions during given movements (i.e., gait, motor tasks)*	35/35	(100)
*Evaluate the appropriateness of the activation among muscles participating to a specific movement (muscle/balance/imbalance/synergy/function)*	34/35	(97)
*Characterize the stretch reflex*	28/35	(80)
*Characterize muscular hyperactivity (e.g., spasticity, spastic co-contraction, spastic dystonia)*	26/33	(79)
5. Utility of sEMG in the definition of an intervention plan - sEMG may be useful when there is need to investigate or quantify: *Abnormalities in the sequential timing of muscular actions during given movements (i.e., gait, motor tasks)*		
*Muscle imbalance/dyssynergia*	26/33	(79)
*Muscular hyperactivity (e.g., spasticity, spastic co-contraction, spastic dystonia)*	25/32	(78)
6. If a therapeutic intervention is administered, sEMG information may prove useful to track changes from baseline in:		
*Sequential timing of muscular actions during given movements (i.e., gait, motor tasks)*	31/35	(89)
*Involuntary muscle activation (e.g., dystonia, ataxia)*	24/32	(75)
7. sEMG assessment can be performed as a stand-alone technique or to complement/optimize other methods used by neurorehabilitation professionals to quantify muscle and physical function. It seems useful adding sEMG to:		
*Gait/motion analysis (with or without motion capture)*	35/35	(100)
*Hyperactivity/Spasticity/muscle tone assessment*	29/34	(85)
*Accelerometry*	25/31	(81)
*Stretch reflex*	26/34	(76)
8. sEMG, when used as biofeedback, may help to:		
*Learn how to change the coordination pattern of an agonist with respect to antagonists and synergists (muscle selectivity)*	30/33	(91)
*Learn how to decrease the activity of overly tense and/or involuntarily hyperactive muscles*	29/33	(88)
*Learn how to increase the activity of weak and/or hypoactive muscles*	29/32	(91)
9.^*^ Professional figure who is most frequently involved in sEMG recordings:		
*Biomedical engineer with a focus on instrumentation, e-health, and rehabilitation*	Ranked 1st	
*Physiotherapist*	Ranked 2nd	
*Kinesiologist/human motion scientist*	Ranked 3rd	
10. Professional figures involved in sEMG signal acquisition, processing, and quality control:		
*Biomedical engineer with a focus on instrumentation, E-health, and Rehabilitation*	31/34	(91)
*Kinesiologist/human motion scientist*	27/34	(79)
11. Professional figures involved in sEMG interpretation:		
*Kinesiologist/human motion scientist*	27/33	(82)
*Clinical neurophysiologist*	26/32	(81)
*Physical Medicine and Rehabilitation physician, also known as physiatrist*	25/32	(78)
*Biomedical engineer with a focus on instrumentation, e-health, and rehabilitation*	25/33	(76)
*Physiotherapist*	24/32	(75)
12. Greater qualification of neurorehabilitation professionals on sEMG would contribute to improve the quality of neurorehabilitation care delivery	28/35	(80)
13. Years of practice/experience with sEMG techniques needed to qualify for providing education and training on the use of sEMG to clinical neurorehabilitation professionals:		
*<1 year: very inadequate*	29/32	(91)
*>5 years: very adequate*	25/29	(86)
14. In addition to basic know-how on sEMG recording (i.e., correct placement of electrodes, adequate skin preparation, etc.), further technical skills are needed:		
*Ability to recognize and filter out artifacts at the skin-electrode interface*	32/35	(91)
*Ability to choose the processing technique that is most appropriate for a given application*	30/35	(86)
15. EMG-derived variables considered of utmost importance for clinical applications in neurorehabilitation:		
*Timing of muscle activations and their variability*	34/34	(100)
*Amplitude estimators (i.e., average rectified value, root mean square)*	28/35	(80)
*Signal quality/reliability indicators (e.g., artifact reporting)*	26/34	(77)
*Envelope time course*	25/33	(76)
16. In addition to knowledge on physiological and non-physiological factors that influence sEMG, neurorehabilitation professionals need further competencies to interpret sEMG:		
*Knowledge about sEMG patterns of recruitment in the main central and peripheral neuromuscular disorders*	33/34	(97)
*Knowledge about the use of sEMG to assess muscular hyperactivity*	31/33	(94)
*Knowledge about sEMG patterns of recruitment of healthy individuals*	31/34	(91)
*Knowledge about the pathologies that affect muscle fiber conduction velocity*	23/30	(77)
17.^*^ Work environment most likely to favor the usage of sEMG:		
*Privately operated clinic (with public or insurance-based reimbursement)*	Ranked 1st	
*Publicly operated clinic (with either public or insurance-based reimbursement)*	Ranked 2nd	
*Privately operated clinic (out-of-pocket)*	Ranked 3rd	
18. Potential barriers to the employment of sEMG in clinical neurorehabilitation:		
*sEMG data analysis/interpretation difficult to perform without specific education/training*	32/33	(97)
*Inadequate education for professionals in neurorehabilitation*	30/34	(88)
*Lack of widely accepted evidence that the use of sEMG improves treatment effectiveness*	26/34	(77)
*Inadequate education and training on sEMG in graduation courses*	27/34	(79)
*Time-consuming*	26/34	(77)

* *Questions were presented as ranking items, with ranking reported in the table only for the first three items*.

**Table 3:** Survey items that did not reach consensus.

**Survey items**	***n***	**(%)**
1. Overall, sEMG is rarely used in clinical neurorehabilitation	19/34	(56)
2. sEMG is currently more relevant for researchers than clinicians	24/34	(71)
3. Regarding the role of sEMG in patient's assessment, sEMG may be useful to:		
*Identify pathological patterns of motor unit behavior*	24/35	(69)
*Evaluate the percent of maximal voluntary activation*	19/35	(54)
*Characterize motor fiber conduction velocity*	22/33	(67)
4. Regarding the utility of sEMG in the definition of an intervention plan, sEMG may be useful when there is need to investigate or quantify:		
*Muscular fatigue*	19/33	(58)
*Abnormalities in the motor unit behavior(muscle/balance/imbalance/synergy/function)*	22/33	(67)
*Abnormalities in the percent of maximal voluntary activation*	16/34	(47)
*Abnormalities in motor fiber conduction velocity*	20/31	(65)
5. If a therapeutic intervention is administered, sEMG information may prove useful to track changes from baseline in:		
*Muscular fatigue*	20/33	(61)
*Muscle imbalance/dyssynergia*	25/34	(74)
*The pattern of motor unit behavior*	20/33	(61)
*The percent of maximal voluntary activation*	16/34	(47)
*Stretch reflex*	22/33	(67)
*Motor fiber conduction velocity*	21/32	(66)
6. sEMG assessment can be performed as a stand-alone technique or to complement/optimize other methods used by neurorehabilitation professionals to quantify muscle and physical function. It seems useful adding sEMG to:		
*Mobility assessment (i.e., Timed Up and Go test; 10-Meter Timed Walk, etc.)*	21/34	(62)
*Muscle strength assessment*	20/35	(57)
*Posture analysis*	19/33	(58)
*Assessment of swallowing*	12/27	(44)
*Tremor analysis*	20/30	(67)
*Goniometric assessments of the joint's passive range of motion*	16/34	(47)
*Goniometric assessment of the joint's active range of motion*	18/34	(53)
*Stand-alone*	18/32	(56)
7. sEMG, when used as biofeedback, may help to:		
*Learn how to associate intrinsic kinesthesia with the desired movement*	16/26	(62)
8. Professional figures involved in sEMG signal acquisition, processing, and quality control:		
*Clinical neurophysiologist*	15/34	(44)
*Kinesiologist/Human motion scientist*	27/34	(79)
9. Professional figures involved in sEMG interpretation:		
*Clinical neurophysiologist*	26/32	(81)
*Neurologist*	11/33	(33)
*Neurophysiopathology/Biomedical laboratory technician*	19/33	(58)
*Occupational therapist*	7/33	(21)
*Speech therapist*	3/32	(9)
10. Greater qualification of clinical neurorehabilitation professionals on sEMG would contribute to reduce the cost of neurorehabilitation care delivery	22/35	(63)
11. Assuming proficiency with sEMG techniques, which of the following professions should provide education and training on the use of sEMG to neurorehabilitation professionals? Please judge the adequacy of the following professional figures:		
*Neurologist*	15/32	(47)
*Neurophysiopathology/Biomedical laboratory technician*	13/32	(41)
*Occupational therapist*	5/32	(16)
*Physical Medicine and Rehabilitation physician, also known as physiatrist*	20/33	(61)
*Speech therapist*	5/29	(17)
12. In addition to basic know-how on sEMG recording (i.e., correct placement of electrodes, adequate skin preparation, etc.), further technical skills are needed:		
*Neurorehabilitation professionals should be able to import EMG data into environments for advanced numerical computing (i.e., MatLab)*	13/35	(37)
13. EMG-derived variables considered of utmost importance for clinical applications in neurorehabilitation:		
*Mean/median envelope*	19/32	(59)
*Normalized envelope (i.e., to maximal voluntary contraction)*	25/34	(74)
*Myoelectric fatigue estimators (i.e., average rectified value and root mean square increase, mean and median frequency reduction)*	20/33	(61)
*Time-frequency / time-scale analysis (wavelet analysis)*	15/33	(45)
*Intensity plot with reference histograms (e.g., control activation timing key)*	15/30	(50)
14. In addition to knowledge on physiological and non-physiological factors that influence sEMG, neurorehabilitation professionals need further competencies to interpret sEMG:		
*Knowledge about myoelectric manifestations of muscle fatigue*	23/32	(72)
*Knowledge about the use of sEMG to assess spasticity*	21/33	(64)
15. Potential barriers to the employment of sEMG in clinical neurorehabilitation:		
*Lack of widely accepted evidence that the use of sEMG in neurorehabilitation impacts the selection of treatments*	24/34	(71)
*Lack of normative ranges to characterize the patient based on sEMG data*	21/34	(62)
*Purchase and maintenance costs of sEMG equipment*	15/34	(44)
*sEMG device/software not clinician-friendly enough*	19/34	(56)
*Uncomfortable for the patient*	2/34	(6)
*No multidisciplinary team available*	23/34	(68)

**1. Current sEMG employment in clinical settings and potential utility (questions 1–10)** – Consensus was reached on that sEMG is more frequently employed in technical/methodological research than clinical research (29/35, 83%), and that it should be used in neurorehabilitation to obtain information on neuromuscular function that is not provided by other assessment techniques/tools (32/35, 91%). With regard to its clinical utility, the respondents agreed by consensus that sEMG can enhance the assessment and characterization of neuromuscular impairments in patients (34/35, 97%), positively influences the intervention plan design (28/35, 80%), allows better tracking of changes in muscle activity from baseline when neurorehabilitation interventions are administered (32/35, 91%), allows evaluating the effects of non-invasive interventions designed to impact muscle activity (such as therapeutic exercise, orthotics, medication, physical agents, manual therapy techniques) (32/35, 91%), and allows evaluating the effects of invasive interventions designed to impact muscle activity (such as surgery and neuromuscular blocks) (30/35, 86%). Moreover, its employment for biofeedback training in case of abnormal patterns of muscle activity that may be modified through motor learning was agreed upon by 30/35 (86%) of the respondents.

When enquired specifically on the role of sEMG in patient's assessment, sEMG was deemed by consensus very likely to be useful to outline the sequential timing of muscular actions during given movements (i.e., gait, motor tasks) (35/35, 100%), evaluate the appropriateness of the muscle activity during a specific movement (muscle balance/imbalance/synergy/function) (34/35, 97.1%), and characterize the stretch reflex (28/35, 80%).

Regarding the utility of sEMG in the definition of an intervention plan, sEMG was indicated as potentially useful when there is need to investigate or quantify abnormalities in the sequential timing of muscular actions during given movements (32/35, 91%), muscle imbalance/dyssynergia (26/33, 79%), and involuntary muscle activity (e.g., dystonia, ataxia) (25/32, 78%). No consensus was reached for 6 of the 8 items questioning whether sEMG information may prove useful to track changes induced by a therapeutic intervention. The cohort agreed that sEMG can, instead, track rehabilitation-induced changes in the sequential timing of muscular actions during given movements (i.e., gait, motor tasks) (31/33, 94%) and for involuntary muscle activation (e.g., dystonia, ataxia) (24/32, 75%).

Regarding the employment of sEMG as a stand-alone technique or in combination with other methods used by neurorehabilitation professionals to assess muscle and physical function, only 18 out of 32 respondents (56%) suggested the stand-alone use of sEMG, whereas they agreed by consensus on the combination of sEMG with gait/motion analysis (with or without motion capture) (35/35, 100%), muscular hyperactivity/muscle tone assessment (29/34, 85%), accelerometry (25/31, 80%), and stretch-reflex assessment (26/34, 77%).

When questioning about the employment of sEMG for biofeedback training in case of abnormal patterns of muscle activity, consensus was reached for its utility in allowing the patient to learn how to “change the coordination pattern of an agonist with respect to antagonists and synergists (muscle selectivity)” (30/33, 91%), “decrease the activity of overly tense and/or involuntarily hyperactive muscles” (29/33, 88%), and “increase the activity of weak and/or hypoactive muscles” (29/32, 91%).

sEMG was expected to have practical utility in clinical neurorehabilitation for five neurological disorders: “Neuromuscular disorders” (31/32, 97%), “Stroke/cerebrovascular diseases” (28/31, 90%), “Spinal cord disorders” (24/29, 83%), “Peripheral nerve disorders” (24/30, 80%), and “Multiple sclerosis/demyelinating diseases” (24/31, 77%).

**Table 4:** Subgroup analysis comparing response rates of clinicians vs. non-clinicians.

**Survey items with significant differences in response rates**	**Percentage of agreement**		**Statistics**	
	**Non-clinicians *N* = 16**	**Clinicians *N* = 19**	**Chi-square**	***p*-value**
- sEMG is currently more relevant for researchers than clinicians	93	53	6.42	0.01
- Regarding the role of sEMG in patient's assessment. sEMG may be useful to evaluate the percent of maximal voluntary activation	44	75	5.11	0.02
- If a therapeutic intervention is administered. sEMG information may prove useful to track changes from baseline in muscular fatigue	47	75	4.02	0.04
- Score the utility of adding sEMG to the following techniques:				
*Accelerometry*	57	90	4.31	0.04
*Goniometric assessment of the joint's active range of motion*	20	71	20.27	<0.0001
*Stand-alone*	27	76	15.80	<0.0001
- To motivate and help patients learning motor strategies that satisfy a particular muscle activity goal, sEMG biofeedback may help them learning how to associate intrinsic kinesthesia with the desired movement.	42	75	5.94	0.01
- Judge the level of involvement of each of the following professionals for sEMG signal acquisition, processing and quality control:				
*Clinical neurophysiologist*	31	53	4.08	0.04
*Physical Medicine and Rehabilitation physician, also known as physiatrist*	20	69	19.22	<0.0001
- Score the level of involvement of each of the following professionals for sEMG interpretation:				
*Occupational therapist*	27	13	4.10	0.04
- Assuming proficiency with sEMG techniques, which of the following professions should provide education and training on the use of sEMG to neurorehabilitation professionals? Please judge the adequacy of the following professional figures:				
*Clinical neurophysiologist*	96	56	6.06	0.01
*Physical Medicine and Rehabilitation physician, also known as physiatrist*	40	81	8.80	0.003
- Based on your experience and knowledge. please score the relevance of the following elements as potential barriers to the clinical use of sEMG:				
*Inadequate education for professionals in neurorehabilitation*	61	94	3.99	0.05
*Purchase and maintenance costs of sEMG equipment*	19	71	21.41	<0.0001

*Non- clinicians: biomedical engineers, kinesiologists/human movement scientists. Clinicians: clinical neurophysiologists; neurophysiopathology/biomedical laboratory technicians; neurologists; Physical Medicine and Rehabilitation physicians, also known as physiatrists; occupational therapists; physiotherapists; speech therapists*.

**2. Professional figures using sEMG and potential advantages of better qualified professionals (questions 11–15)** – Interviewees were asked to rank the professional figures who are most frequently involved in sEMG recordings in neurorehabilitation settings. Respondents listed “Biomedical engineer” in first place (13, 3, and 4 respondents for 1st, 2nd, and 3rd place, respectively) followed by “Physiotherapist” (6, 5, and 4 respondents for 1st, 2nd, and 3rd place, respectively) and “Kinesiologist/Human movement scientist” (3, 5, and 6 respondents for 1st, 2nd, and 3rd place, respectively).

When judging the level of involvement of each of the professionals for sEMG signal acquisition, processing and quality control, consensus was reached only for “Biomedical engineer” (32/34, 94%) and “Kinesiologist/Human movement scientist” (27/34, 79%) but not for other professions. When asked about the involvement for sEMG interpretation, respondents agreed by consensus on five professional figures: “Kinesiologist/Human movement scientist” (27/33, 82%), “Clinical neurophysiologist” (26/32, 81%), “PM&R” (25/32, 78%), “Biomedical engineer” (25/33, 76%), and “Physiotherapist” (24/32, 75%). Both PM&R doctors and physiotherapists were professionals with the highest number of “very high” agreement (23/32, 41%).

Participants were also asked whether greater qualification of neurorehabilitation professionals on sEMG would contribute to improve the quality of neurorehabilitation care and reduce the cost of its delivery. Consensus was reached for quality improvement (28/35, 80%) but not for cost reduction (22/35, 63%).

**3. Education, training, and teaching (questions 16–20)** – Regarding the adequacy of the professional figures in the education, training, and teaching of neurorehabilitation professionals on sEMG, consensus (i.e., adequate to very adequate) was reached for four figures: “Biomedical engineer” (29/32, 91%), “Kinesiologist/Human movement scientist” (25/32, 78%), “Physiotherapist” (25/33, 76%), and “Clinical neurophysiologist” (24/32, 75%). A combination of professional figures was also indicated as adequate (27/32, 84%). Interestingly, this option received the largest number of “very adequate” preferences (21/32, 66%), followed by “Biomedical engineer” (14/32, 44%), and “Physiotherapist” (12/33, 36%).

Participants were asked to indicate how many years of practice/experience with sEMG techniques are necessary to qualify for providing education and training on the use of sEMG for clinical neurorehabilitation. They agreed by consensus that practice/experience <1 year is inadequate to very inadequate (29/32, 91%). At least 5 years were indicated as an adequate to very adequate period (25/29, 86%).

When analyzing the technical skills for sEMG recording and interpretation that neurorehabilitation professionals should own (in addition to basic know-how on sEMG recording, such as correct placement of electrodes, adequate skin preparation, etc.), respondents agreed they should be able to recognize and filter out artifacts at the skin-electrode interface (i.e., baseline noise contamination, movement artifacts, cross-talk, etc.) (32/35, 91%), and choose the processing technique that is most appropriate for a given application (30/35, 86%).

With regard to the importance of sEMG-derived variables for clinical applications, consensus was reached for the “Timing of muscle activation and their variability” (34/34, 100%), amplitude estimators (i.e., average rectified value, root mean square) (28/35, 80%), “Signal quality/reliability indicators (e.g., artifact reporting)” (26/34, 77%), and envelope time course (25/33, 76%).

When asked to indicate further competencies to complement basic knowledge on physiological and non-physiological factors that influence sEMG, respondents agreed by consensus that neurorehabilitation professionals should own knowledge about sEMG patterns of recruitment in the main central and peripheral neuromuscular disorders (33/34, 97%), about the use of sEMG to assess muscular hyperactivity (31/33, 94%), about sEMG patterns of recruitment of healthy individuals (31/34, 91%), and about the pathologies affecting muscle fiber conduction velocity (23/30, 77%).

**4. Potential barriers to sEMG usage (questions 21 and 22)** – Respondents agreed by consensus on 5 of the 12 suggested potential barriers limiting the widespread use of sEMG in clinical neurorehabilitation: “sEMG data analysis/interpretation is difficult to perform without specific education/training” (32/33, 97%), “Inadequate education for professionals in neurorehabilitation” (30/34, 88%), “Lack of widely accepted evidence that the use of sEMG improves treatment effectiveness” (26/34, 77%), “Inadequate education and training on sEMG in graduation courses” (27/34, 79%), and “Time-consuming” (26/34, 76%).

We asked participants to rank which clinical environment is most likely to favor the usage of sEMG. Respondents listed “Privately operated clinic (with public or insurance-based reimbursement)” in first place (ranked 1st by 7 respondents; 2nd by 11 respondents; 3rd by 8 respondents) followed by “Publicly operated clinic (with either public or insurance-based reimbursement)” (ranked 1st by 10 respondents; 2nd by 5 respondents; 3rd by 4 respondents) and “Privately operated clinic (out-of-pocket)” (ranked 1st by 1 respondent; 2nd by 8 respondents; 3rd by 14 respondents).

Table 4 reports the results of the subgroup analyses carried out to verify whether responses were influenced by the professional figure of the interviewees. Significant differences were detected in the response rates of non-clinicians and clinicians regarding a number of items. Among these, clear discrepancies emerged in the way sEMG is viewed (“more relevant for researchers than clinicians” for 93 and 53% of non-clinicians and clinicians, respectively, *p* = 0.01), and in a potential barrier perceived to limit its clinical translation (“Purchase and maintenance costs of sEMG equipment”), which was deemed relevant by 71% of the clinicians and by 19% of the non-clinicians (*p* < 0.0001).

## Discussion

We conducted survey research to elucidate key aspects on four main themes including trends in the current employment of sEMG in clinical settings and its potential utility, professional figures involved in sEMG assessment and interpretation, educational aspects, and potential barriers to the incorporation of this technique in daily neurorehabilitation practice. By building consensus, we intended to gather information from experts in the field who published prominently on the topic for establishing a common platform to streamline future clinically-applied research on the topic. Importantly, the majority of the interviewed cohort were clinicians (57%). Consensus was reached on 17 of the 22 proposed questions encompassing the four main themes of the survey focused.

### Current Usage and Perceived Clinical Utility of sEMG

Contrary to our first assumption, no consensus was reached on the statement that sEMG is rarely used in clinical neurorehabilitation (56%). This finding is in disagreement with previous reports (2–5), which, overall, outlined a negative scenario where methodological and clinical knowledge is widely available but clinicians fail to access and use sEMG in daily practice. A possible reason for such discrepancy may reside in the different professional backgrounds and countries of the interviewees [only clinicians from USA in Feldner's study (5) vs. professionals with wide-ranging background mostly from Europe in our survey]. The item on how frequently sEMG is employed in neurorehabilitation was extensively commented upon. Experts highlighted that employing sEMG is not a matter of frequency but utility and appropriateness, i.e., using a device/technique/therapeutic approach only when it should be used and vice versa. In this regard, the invitees agreed by consensus on the appropriateness and utility of sEMG for neurorehabilitation purposes.

sEMG was deemed by consensus to provide unique information on neuromuscular function that is not offered by other assessment techniques/tools. Among the advantages for which consensus was established (ranging from 80 to 97% of the respondents, see questions 5–10), its use was considered to substantially enhance the quality of patient's assessment. This result supports the use of sEMG as a tool for motion analysis as it can record and quantify clinically important muscle-related activity. Given that sEMG is deemed to cause minimal burden to the subject or the patient (1), this result highlights the importance of collecting such muscle-level information from patients in neurorehabilitation settings.

Participants also agreed that sEMG has a role in the assessment of psychophysical indicators of reaction and movement time in clinical settings as well as to evaluate gait and posture. Consensus was not reached (67%) on whether it could be used to differentiate the many types of tone-regulations impairments such as myoclonus, dystonia, and tremors. It may be that these latter applications require more clinical validation before being employed in neurorehabilitation settings.

As strongly agreed by the respondents, the potential of sEMG for accurate analysis of movement disorders is maximized by its combination with other quantitative and qualitative methods, such as gait/motion analysis, muscular hyperactivity/reflex/tone assessments, and accelerometry. Interestingly, the item on muscular hyperactivity was widely commented on. Two invitees for example, remarked that sEMG could differentiate between spasticity and rigidity, as suggested by Levin et al. (27), Mullick et al. (28), and more recently by Baude et al. (29). Moreover, regardless of whether sEMG is applied stand-alone or combined with other techniques/tools, according to the cohort's aggregate view (86–91%), it can specifically track objective changes from baseline following therapeutic interventions in the sequential timing of muscular actions during given movements (i.e., gait, motor tasks) and for involuntary muscle activation (e.g., dystonia, ataxia).

The cohort was also very clear in highlighting by consensus that sEMG is more frequently employed in technical/methodological reports than in clinical research, possibly suggesting that a substantial distance still exists between researchers and clinicians, in agreement with previous reports (2, 3, 5, 18, 19, 22). A possible explanation for such a gap might be a limited ability to transfer research knowledge to practice, requiring clinical translational research, which is still limited. Moreover, it is also likely that clinicians do not keep updated with research findings, which corroborates historical claims that clinicians fail to access and thus employ EBP information into daily practice despite the vast availability of excellent resources (30). According to Berwick (31), “Health care is rich in evidence-based innovations, yet even when such innovations are implemented successfully in one location, they often disseminate slowly, if at all.” Also, rehabilitation professionals now have easy access to evidence syntheses, systematic reviews, and meta-analyses that condense research articles, still EBP-derived knowledge hardly and slowly transfers to patient's bedside (7, 32). Even if not tested by the present study, we speculate that slow dissemination of EBP-derived knowledge may also apply to the persisting resistance to sEMG employment despite decades of development of this technology (33, 34), and a massive body of knowledge accumulated so far in support of its translational utility. Enhancing education and training (i.e., by including technology teaching into undergraduate and postgraduate physiotherapy/health professional courses and in medical courses) as well as increasing clinicians' awareness of the potential advantages offered by this technology may help accelerate the transfer from proven health care discoveries to patient care needs. Moreover, in the attempt to fill the gap, we believe that professional and clinicians' associations (e.g., the American Physical Therapy Association) could serve as cultural bonds between researchers and practitioners and ensure the dissemination of novel knowledge.

### Potential Barriers and Educational Aspects

Specific education and training of professionals were considered to play a decisive role, particularly to improve the quality of neurorehabilitation care. Accordingly, inadequate education and training on sEMG in graduate courses, lack of continuing education for professionals in neurorehabilitation and time-consuming set up were listed as the main barriers to the clinical employment of this technique.

Acquiring and consolidating knowledge on physiological and non-physiological factors that influence sEMG was also put forward as a key point for the interpretation of sEMG findings by neurorehabilitation professionals. In particular, strong consensus (>90%) was achieved for three elements to be part of their educational background: (1) knowledge on the sEMG patterns of recruitment of healthy individuals, (2) knowledge on the main central and peripheral neuromuscular disorders, and (3) salient sEMG features of muscles affected by spasticity/hyperactivity. Relatedly, the respondents agreed that sEMG data analysis and interpretation may be difficult to perform without such knowledge and specific training, thus confirming the key role of education in the gap between EBP and daily practice. The cohort of experts agreed that professions teaching sEMG must have a minimum of 5 years of experience with sEMG, which may be due to the lack of teaching in academic courses and the need for learning by experience or by trial and error. While this period could undeniably appear very long, it may be necessary for those professional engaged in sEMG teaching.

Interestingly, even though the cohort was mainly composed of clinicians, the professional figures who were deemed best qualified for teaching sEMG to neurorehabilitation professionals were not medical doctors nor physiotherapists. Indeed, biomedical engineers and kinesiologists/movement scientists were ranked, respectively, in first and second place, provided that they hold superior expertise and skills which, regardless of the professional figure, was indicated as the key factor.

The item on potential barriers was extensively commented upon, with invitees converging on four additional factors: (1) poor reliability/validity if the subcutaneous tissue layer is too thick, which may often be the case in the adult and aged (sedentary) population, and particularly in women, even though the role of fat interference has also been downplayed (2, 35) general distrust in technology; (3) lack of self-confidence due to poor education; (4) need of a dedicated team.

Taking together the interviewees' responses and comments on factors potentially limiting the usage of sEMG in neurorehabilitation, it appears that the integration of sEMG into an agreed framework for diagnosis and treatment is still challenging for clinicians. Furthermore, there is currently poor translational evidence to make sEMG part of a coherent diagnostic or measurement context and to use it to track the right clinical outcomes. For all these reasons, it is likely that sEMG is not used extensively in neurorehabilitation.

### Professional Figures Involved in sEMG

As for teaching, biomedical engineers were deemed the professional figure most frequently involved in sEMG signal acquisition, processing, and quality control. Kinesiologists/human movement scientists, clinicians (clinical neurophysiologists, PM&R physicians, physiotherapists) and biomedical engineers were, instead, indicated as those most likely in charge of data analysis, post-processing, features extraction etc. Among the comments accompanying this specific item, the relatively new professional figure of “human motion analyst” was put forward by some respondents as a possible reference to manage sEMG assessments in the clinical setting.

### Technical and Methodological Aspects

Data indicate that neurorehabilitation professionals should hold basic know-how (i.e., correct placement of electrodes, adequate skin preparation, etc.) but also additional abilities to recognize and filter out artifacts, distinguish between cross-talk and co-activation, and also choose the most appropriate processing technique. More advanced expertise, such as importing data into external environments and further computing were regarded as “not required for all clinical neurorehabilitation professionals.” Overall, the item about the ideal technical skills was also widely commented. What clearly emerged from additional reasoning and elaborations from the experts was that a basic knowledge about the identification of “bad” signals (e.g., power line interference, artifacts, poor contacts) may be sufficient for clinicians, whereas familiarity with signal acquisition issues management (data clipping, low-pass and high-pass filtering, down-over sampling, algorithms for the estimation of signal-to-noise ratio, etc.) along with relatively more advanced analyses (e.g., power spectral density, developing custom software scripts, etc.) are skills that are necessary for professionals working in the laboratory and dealing with human motion analysis.

A question specifically surveyed which sEMG variables should be considered. This is a relevant point considering the “*too many data*–*no data*” paradox (36), which refers to the difficulties that clinicians experience in extracting clinically meaningful parameters from newly introduced biomedical technologies (e.g., sEMG, gait analysis, back shape measurement), for which a general sentiment of distrust is often shown by clinical practitioners. Four sEMG-based parameters were indicated as important to report: (1) timing of muscle activations, (2) amplitude estimators, (3) envelope time course, and (4) indicators of signal quality/reliability. From experts' free commenting, power spectrum density, and muscle fiber conduction velocity also emerged as likely important to control for data quality and reliability.

### Differences in Responses Between Clinicians and Non-clinicians

Data revealed significantly different views between clinicians (54% of the cohort) and non-clinicians (46%) for several issues. For instance, unlike clinicians, the majority of the non-clinicians considered sEMG more relevant for research than clinical purposes. As for those factors potentially limiting the usage of sEMG in clinical practice, different beliefs emerged between the two categories being “inadequate education” and “purchase and maintenance costs of sEMG equipment” perceived as more relevant barriers by clinicians. These findings reveal that for selected items the professional figure background influence the questionnaire responses.

## Limitations

The findings of the present survey research may be limited by the characteristics and geographical location of the selected contributors who participated in the survey, possibly subjecting the survey findings to selection bias as the usage of sEMG may vary by country. In particular, 86% of the interviewees were from Europe and 54% from Italy, possibly reflecting a greater sensitivity and interest to the problem in this country. Although we collected responses from the majority (67%) of the sEMG experts invited, it cannot be excluded that responses from a larger number of individuals with different backgrounds may have led to different results, possibly leading to reduced likelihood of consensus. Another element that needs to be considered in the interpretation of the findings is the choice to interview only scientists, researchers, and clinicians who had authored indexed articles on the topic rather than clinicians who did not. Therefore, the results of the survey and their external validity and generalizability may be limited and biased by this choice and will need to be compared to a larger sample of clinicians who specifically engage in neurorehabilitation on a daily basis, but not necessarily on research. Moreover, while being iterative in the conception and planning of the survey, we acknowledge that the methodological robustness of this study could have been enhanced by adding one or more rounds to the present one-round survey.

Finally, while we attempted to be comprehensive in the development of the survey questions and sub-items, other questions could have been asked to address specific issues that were here possibly overlooked.

## Concluding Remarks

This survey research clarified several aspects of sEMG in neurorehabilitation ranging from current trends in its use, educational, technical, and methodological features as well as the translational outreach and potential utility of this technique for clinicians and patients.

In particular, sEMG was indicated as practically useful in clinical neurorehabilitation for patient assessment, to define the intervention plan and complement/optimize other methods used to quantify muscle and physical function. Nevertheless, the aggregate opinion of the interviewed experts clearly revealed that sEMG is more frequently employed in technical/methodological than clinical research. Moreover, the slow dissemination of research findings, lack of education on sEMG and lack of incorporation of the patients' goals when applying the technology seem to prevent prompt translation into practice. Additionally, multidisciplinary competences are necessary to face the challenges of the complexity of the matter, beside the identification of more specific procedures, for increasing clinical use and benefits for the patients.

Future translational studies aimed at testing whether the addition of sEMG brings clinically important benefits are required to fill the gap between research and clinical practice, which may itself limit the employment of sEMG in neurorehabilitation. With the present survey findings obtained by bringing together the expertise, guidance, and insights of leading experts in the field, we are now better positioned to open a debate on the appropriateness and value of sEMG for neurorehabilitation professionals and its potential translation into clinical practice.

## Data Availability Statement

All datasets generated for this study are included in the article/Supplementary Material.

## Ethics Statement

The studies involving human participants were reviewed and approved by Institutional Review Board Department of Biomedical Sciences University of Sassari, Italy. The patients/participants provided their written informed consent to participate in this study.

## Author Contributions

AMa, AC, and FD conceived the idea and managed all aspects of the work. LB-O, AB, UD, MK, NM, DM, AMe, SR, and AT developed the survey questions, developed and piloted the survey. All authors provided critical feedback and helped shape the research, analysis, and manuscript. All authors contributed to the article and approved the submitted version.

## Conflict of Interest

The authors declare that the research was conducted in the absence of any commercial or financial relationships that could be construed as a potential conflict of interest.
