# Supplementary material for: A Survey on the Use and Barriers of Surface Electromyography in Neurorehabilitation
Source: Front Neurol. 2020 Oct 2;11:573616. doi: 10.3389/fneur.2020.573616 (PMC7566898; doi:10.3389/fneur.2020.573616)
Supplement: Supplementary file 1 [file Data_Sheet_1.docx]

**DELPHI PROCESS ON THE USE OF SURFACE ELECTROMYOGRAPHY IN NEUROREHABILITATION**

**International consensus on the clinical use of surface electromyography in neurorehabilitation settings for assessments and decision-making:**

**current applications, prospective applications and barriers**

Clinical professional figures operating in the field of neurorehabilitation are facing growing demands to validate their clinical observations and techniques with objective data. Recording the electrical activation of muscle fibers through surface electrodes positioned on the skin over the muscle/s of interest (surface electromyography, sEMG) may be of help to this objective since it provides quantitative information about the timing or magnitude of muscle activity when assessing muscle excitation or patterns of movement.

*Please, answer the questions below. If the content of an item falls outside your specific expertise, so that you would feel uncomfortable or inadequate to provide an answer, please leave the item unanswered (consensus will be determined only from answered items). Please, also specify the reason/s for non-response in the “Other comments” section below the unanswered item.*

1. Overall, sEMG is rarely used in clinical neurorehabilitation.

Please score your level of agreement with this statement.

| Strongly  disagree |  | Neutral |  | Definitely  agree |
| --- | --- | --- | --- | --- |
| O | O | O | O | O |

Other comments _______________________________________________

2. sEMG is more frequently employed in technical/methodological research than clinical research.

Please score your level of agreement with this statement.

| Strongly  disagree |  | Neutral |  | Definitely  agree |
| --- | --- | --- | --- | --- |
| O | O | O | O | O |

Other comments _______________________________________________

3. Based on your knowledge and experience, is sEMG currently more relevant for researchers than for clinicians?

| Definitely  not |  | Neutral |  | Definitely  yes |
| --- | --- | --- | --- | --- |
| O | O | O | O | O |

Other comments _______________________________________________

4. sEMG provides information on neuromuscular function that is not provided by other assessment techniques/tools in neurorehabilitation.

Please rate the level of agreement with this statement.

| Definitely  disagree |  | Neutral |  | Definitely  agree |
| --- | --- | --- | --- | --- |
| O | O | O | O | O |

Other comments _______________________________________________

5. There is ongoing debate on the practical utility of sEMG in clinical neurorehabilitation. sEMG information on neuromuscular activation may:

|  | Very  unlikely |  | Neutral |  | Very  likely |
| --- | --- | --- | --- | --- | --- |
| enhance the assessment and characterization of neuromuscular impairments  in patients | O | O | O | O | O |
| influence the intervention plan design | O | O | O | O | O |
| allow to better track the changes in muscle activity from baseline  when neurorehabilitation interventions are administered | O | O | O | O | O |
| allow to evaluate the effects of non-invasive interventions designed to impact muscle activity (such as therapeutic exercise, orthotics, medication, physical agents, manual therapy techniques) | O | O | O | O | O |
| allow to evaluate the effects of invasive interventions designed to impact muscle activity (such as surgery and neuromuscular blocks) | O | O | O | O | O |
| be employed as biofeedback training if the clinician identifies abnormal  patterns of muscle activity that may be modified through motor learning. | O | O | O | O | O |

Other uses of sEMG not listed above _______________

6. Regarding the role of sEMG in patient’s assessment, sEMG may be useful to:

|  | Very  unlikely |  | Neutral |  | Very  likely |
| --- | --- | --- | --- | --- | --- |
| outline the sequential timing of muscular actions during given movements (i.e. gait, motor tasks) | O | O | O | O | O |
| evaluate muscular fatigue | O | O | O | O | O |
| evaluate the appropriateness of the activation among muscles participating to a specific movement (muscle balance/imbalance/synergy/function) | O | O | O | O | O |
| identify pathological patterns of motor unit behavior | O | O | O | O | O |
| evaluate the percent of maximal voluntary activation | O | O | O | O | O |
| characterize the stretch reflex | O | O | O | O | O |
| characterize other involuntary muscle activation (e.g. dystonia, ataxia) | O | O | O | O | O |
| characterize motor fiber conduction velocity | O | O | O | O | O |

Other uses of sEMG as an assessment tool in clinical neurorehabilitation not listed above _______________

Other comments __________________________________________________________________

7. Regarding the utility of sEMG in the definition of an intervention plan, sEMG may be useful when there is need to investigate or quantify:

|  | Not useful  at all |  | Neutral |  | Very  useful |
| --- | --- | --- | --- | --- | --- |
| abnormalities in the sequential timing of muscular actions during given movements (i.e. gait, motor tasks) | O | O | O | O | O |
| muscular fatigue | O | O | O | O | O |
| muscle imbalance/dissynergy | O | O | O | O | O |
| abnormalities in the motor unit behavior | O | O | O | O | O |
| abnormalities in the percent of maximal voluntary  activation | O | O | O | O | O |
| abnormalities in the  stretch reflexes | O | O | O | O | O |
| involuntary muscle activation (e.g. dystonia, ataxia) | O | O | O | O | O |
| abnormalities in motor fiber conduction velocity | O | O | O | O | O |

Other uses of sEMG not listed above _______________

8. If a therapeutic intervention is administered, sEMG information may prove useful to track changes from baseline in:

|  | Very  unlikely |  | Neutral |  | Very  likely |
| --- | --- | --- | --- | --- | --- |
| sequential timing of muscular actions during given movements  (i.e. gait, motor tasks) | O | O | O | O | O |
| muscular fatigue | O | O | O | O | O |
| muscle imbalance/dissinergy | O | O | O | O | O |
| the pattern of motor unit behavior | O | O | O | O | O |
| the percent of maximal voluntary activation | O | O | O | O | O |
| stretch reflex | O | O | O | O | O |
| involuntary muscle activation (e.g. dystonia, ataxia) | O | O | O | O | O |
| motor fiber conduction velocity | O | O | O | O | O |

Other uses of sEMG not listed above _______________

9. sEMG assessment can be performed as a stand-alone technique or to complement/optimize other methods used by neurorehabilitation professionals to quantify muscle and physical function.

Please score the utility of adding sEMG to the following techniques:

|  | Not at all useful |  | Neutral |  | Very useful |
| --- | --- | --- | --- | --- | --- |
| Accelerometry | O | O | O | O | O |
| Gait/motion analysis (with or without motion capture) | O | O | O | O | O |
| Mobility assessment (i.e. TUG, 10MTW, etc.) | O | O | O | O | O |
| Stretch reflex | O | O | O | O | O |
| Muscle strength assessment | O | O | O | O | O |
| Posture analysis | O | O | O | O | O |
| Spasticity/muscle tone assessment | O | O | O | O | O |
| Assessment of swallowing | O | O | O | O | O |
| Tremor analysis | O | O | O | O | O |
| Goniometric assessments of the joint’s passive range of motion | O | O | O | O | O |
| Goniometric assessment of the joint’s active range of motion | O | O | O | O | O |
| Stand-alone | O | O | O | O | O |

*Abbreviations: TUG, Timed-Up-and-Go; Ten-Meter Timed Walk.*

Other scenarios to be considered__________________________________________________

Other comments _______________________________________________

10. To motivate and help patients learning motor strategies that satisfy a particular muscle activity goal, sEMG biofeedback may help them:

|  | Very  unlikely |  | Neutral |  | Very  likely |
| --- | --- | --- | --- | --- | --- |
| learning how to change the coordination pattern of an agonist with  respect to antagonists and synergists (muscle selectivity) | O | O | O | O | O |
| learning how to decrease the activity of overly tense and/or involuntarily  hyperactive muscles | O | O | O | O | O |
| learning how to increase the activity of weak and/or hypoactive muscles | O | O | O | O | O |
| learning how to associate intrinsic kinesthesia with the desired movement | O | O | O | O | O |

Other comments _______________________________________________

11. In neurorehabilitation settings, which is the professional figure who is most frequently involved in sEMG recordings?

Please rank the following professional figures from the most likely to least likely. You can adjust the rank by dragging/dropping each option.

| ⁞ | ↨ | Biomedical engineer with a focus on  instrumentation, E-health, and Rehabilitation |
| --- | --- | --- |
| ⁞ | ↨ | Clinical neurophysiologist |
| ⁞ | ↨ | Kinesiologist/Human movement scientist/Sport scientist/Exercise scientist |
| ⁞ | ↨ | Neurologist |
| ⁞ | ↨ | Neurophysiopathology/Biomedical laboratory technician |
| ⁞ | ↨ | Occupational therapist |
| ⁞ | ↨ | Physical Medicine and Rehabilitation physician, also known as physiatrist |
| ⁞ | ↨ | Physiotherapist |
| ⁞ | ↨ | Speech therapist |

Other figures____________________________

Other comments_______________________________________________

12. Please judge the level of involvement of each of the following professionals for sEMG signal acquisition, processing and quality control

|  | Very low |  | Neutral |  | Very  high |
| --- | --- | --- | --- | --- | --- |
| Biomedical engineer with a focus on  instrumentation, E-health, and Rehabilitation | O | O | O | O | O |
| Clinical neurophysiologist | O | O | O | O | O |
| Kinesiologist/Human movement scientist/Sport scientist/Exercise scientist | O | O | O | O | O |
| Neurologist | O | O | O | O | O |
| Neurophysiopathology/Biomedical laboratory  technician | O | O | O | O | O |
| Occupational therapist | O | O | O | O | O |
| Physical Medicine and Rehabilitation physician,  also known as physiatrist | O | O | O | O | O |
| Physiotherapist | O | O | O | O | O |
| Speech therapist | O | O | O | O | O |

Other figures____________________________

Other comments_______________________________________________

13. Please judge the level of involvement of each of the following professionals for sEMG interpretation.

|  | Very low |  | Neutral |  | Very high |
| --- | --- | --- | --- | --- | --- |
| Biomedical engineer with a focus on  instrumentation, E-health, and Rehabilitation | O | O | O | O | O |
| Clinical neurophysiologist | O | O | O | O | O |
| Kinesiologist/Human movement scientist/Sport scientist/Exercise scientist | O | O | O | O | O |
| Neurologist | O | O | O | O | O |
| Neurophysiopathology/Biomedical laboratory technician | O | O | O | O | O |
| Occupational therapist | O | O | O | O | O |
| Physical Medicine and Rehabilitation physician, also known as physiatrist | O | O | O | O | O |
| Physiotherapist | O | O | O | O | O |
| Speech therapist | O | O | O | O | O |

Other figures____________________________

Other comments_______________________________________________

14. Assuming that sEMG is a useful tool in clinical neurorehabilitation, would greater qualification of neurorehabilitation professionals on sEMG contribute towards improving the quality of neurorehabilitation care delivery?

| Definitely not |  | Neutral |  | Definitely  yes |
| --- | --- | --- | --- | --- |
| O | O | O | O | O |

Other comments _______________________________________________

15. Would greater qualification of clinical neurorehabilitation professionals on sEMG contribute to reduce the cost of neurorehabilitation care delivery?

| Definitely not |  | Neutral |  | Definitely  yes |
| --- | --- | --- | --- | --- |
| O | O | O | O | O |

Other comments _______________________________________________

16. Given proficiency with the EMG techniques, which of the following professions should provide education and training on the use of sEMG to neurorehabilitation professionals?

Please judge the adequacy of the following professional figures:

|  | Inadequate |  | Neutral |  | Very adequate |
| --- | --- | --- | --- | --- | --- |
| Biomedical engineer with a focus on  instrumentation, E-health, and Rehabilitation | O | O | O | O | O |
| Clinical neurophysiologist | O | O | O | O | O |
| Kinesiologist/Human movement scientist/Sport scientist/Exercise scientist | O | O | O | O | O |
| Neurologist | O | O | O | O | O |
| Neurophysiopathology/Biomedical laboratory  technician | O | O | O | O | O |
| Occupational therapist | O | O | O | O | O |
| Physical Medicine and Rehabilitation physician, also known as physiatrist | O | O | O | O | O |
| Physiotherapist | O | O | O | O | O |
| Speech therapist | O | O | O | O | O |
| A combination of these professions | O | O | O | O | O |

Other figures _______________________________________________

In case a combination of professions was chosen, please specify your best rank _____________________________

Other comments _______________________________________________

17. How many years of practice/experience with sEMG techniques are necessary to qualify for providing education and training on the use of sEMG to clinical neurorehabilitation professionals?

|  | Inadequate |  | Neutral |  | Very adequate |
| --- | --- | --- | --- | --- | --- |
| <1 years | O | O | O | O | O |
| 1-2 years | O | O | O | O | O |
| 3-5 years | O | O | O | O | O |
| >5 years | O | O | O | O | O |

Other comments _______________________________________________

18. In addition to basic know-how on sEMG recording (i.e. correct placement of electrodes, adequate skin preparation, etc.), further technical skills may help clinical neurorehabilitation professionals in the analysis and interpretation of sEMG signals.

Please state your level of agreement with the following statements:

|  | Strongly  disagree |  | Neutral |  | Definitely  agree |
| --- | --- | --- | --- | --- | --- |
| Neurorehabilitation professionals should be able to recognize and filter out artefacts at the skin-electrode interface (i.e. baseline noise contamination, movement artefacts), cross-talk, etc. | O | O | O | O | O |
| Neurorehabilitation professionals should be able to choose the processing  technique that is most appropriate for a given application | O | O | O | O | O |
| Neurorehabilitation professionals should be able to import EMG data into  environments for advanced numerical computing (i.e. MatLab) | O | O | O | O | O |

Other technical skills to be considered__________________________________________________

Other comments____________________________________________________________________

19. What are the EMG-derived variables you consider most important for clinical applications?

|  | | Strongly  disagree | |  | | Neutral | |  | | Definitely  agree | |
| --- | --- | --- | --- | --- | --- | --- | --- | --- | --- | --- | --- |
| Amplitude estimators (ARV, RMS, …) | | O | | O | | O | | O | | O | |
| Envelope time course | | O | | O | | O | | O | | O | |
| Mean/Median Envelope | | O | | O | | O | | O | | O | |
| Normalized Envelope (to MVC, MMT) | | O | | O | | O | | O | | O | |
| Myoelectric fatigue estimators (RMS e ARV increase, MNF e MDF reduction) | | O | | O | | O | | O | | O | |
| Timing of muscle activations and their variability | | O | | O | | O | | O | | O | |
| Time-frequency / time- scale analysis (wavelet analysis) | | O | | O | | O | | O | | O | |
| Intensity plot with reference histograms (e.g. control activation timing key) | | O | | O | | O | | O | | O | |
| Signal quality / signal reliability indicators (e.g. artifact reporting) | | O | | O | | O | | O | | O | |

*Abbreviations:* *ARV, average rectified value; MEDF, median frequency; MNF, mean frequency; MVC, maximal voluntary contraction; RMS, root mean square.*

Other techniques to be considered__________________________________________________

Other comments_______________________________________________

20. In addition to knowledge on physiological and non-physiological factors that influence sEMG, neurorehabilitation professionals need further competencies to interpret sEMG.

Please state your degree of agreement on the relevance of the following competences:

|  | Not  relevant |  | Neutral |  | Highly  relevant |
| --- | --- | --- | --- | --- | --- |
| Knowledge about sEMG patterns of recruitment of healthy  individuals | O | O | O | O | O |
| Knowledge about sEMG patterns of recruitment in the main  central and peripheral neuromuscular disorders | O | O | O | O | O |
| Knowledge about which pathologies affect muscle fiber conduction velocity | O | O | O | O | O |
| Knowledge about myoelectric manifestations of muscle fatigue | O | O | O | O | O |
| Knowledge about the use sEMG to assess spasticity | O | O | O | O | O |

Other competencies to be considered__________________________________________________

Other comments _______________________________________________

21. Based on your knowledge and experience, please rank which work environment is most likely to least likely to favor the usage of sEMG. You can adjust the rank by dragging/dropping each option.

| ⁞ | ↨ | Privately operated clinic (out-of-pocket) |
| --- | --- | --- |
| ⁞ | ↨ | Privately operated clinic (with public or insurance-based reimbursement) |
| ⁞ | ↨ | Publicly operated clinic (with either public or insurance-based reimbursement) |
| ⁞ | ↨ | Others |
| ⁞ | ↨ | No difference among the cited work environments |

Other environments (please specify) ________________________________

Other comments_______________________________________________

22. Several factors may concur to limit the widespread usage of sEMG in clinical neurorehabilitation.

Based on your experience and knowledge, please score the relevance of the following elements as potential barriers to the clinical use of sEMG.

|  | Not  relevant  at all |  | Neutral |  | Very  relevant |
| --- | --- | --- | --- | --- | --- |
| Lack of widely accepted evidence that the use of sEMG in  neurorehabilitation impacts the selection of treatments | O | O | O | O | O |
| Lack of widely accepted evidence that the use of sEMG improves  treatment effectiveness | O | O | O | O | O |
| Lack of normative ranges to characterize the patient based on  sEMG data | O | O | O | O | O |
| Inadequate education for professionals in neurorehabilitation | O | O | O | O | O |
| Inadequate education and training on sEMG in graduation courses | O | O | O | O | O |
| Limited relevance of sEMG as a clinical tool | O | O | O | O | O |
| Purchase and maintenance costs of sEMG equipment | O | O | O | O | O |
| sEMG data analysis/interpretation difficult to perform  without specific education/training | O | O | O | O | O |
| sEMG device/software not clinician-friendly enough | O | O | O | O | O |
| Time-consuming | O | O | O | O | O |
| Uncomfortable for the patient | O | O | O | O | O |
| No multidisciplinary team available | O | O | O | O | O |

Other potential barriers_____________________________________________________

Other comments_______________________________________________

23. Please indicate your geographical origin:

| Africa | O |
| --- | --- |
| Asia | O |
| Oceania | O |
| Europe | O |
| North America | O |
| South America | O |

Please specify your country of origin ____________________________________

24. Please indicate the geographical area where you received your first academic education:

| Africa | O |
| --- | --- |
| Asia | O |
| Oceania | O |
| Europe | O |
| North America | O |
| South America | O |

Please specify the country ____________________________________

25. Please indicate the geographical area where you received your advanced academic education (if applicable):

| Master’s degree | Africa | O |
| --- | --- | --- |
|  | Asia | O |
|  | Oceania | O |
|  | Europe | O |
|  | North America | O |
|  | South America | O |

Please specify the scientific field ____________________________________

| Doctor of Philosophy (Ph.D.) | Africa | O |
| --- | --- | --- |
|  | Asia | O |
|  | Oceania | O |
|  | Europe | O |
|  | North America | O |
|  | South America | O |

Please specify the scientific field ____________________________________

26. In which continent do you currently live and work?

| Africa | O |
| --- | --- |
| Asia | O |
| Oceania | O |
| Europe | O |
| North America | O |
| South America | O |

Please specify the country ____________________________________

27. Thinking about your professional activity, which of the labels best describes you?

|  | Yes | No |
| --- | --- | --- |
| Researcher | O | O |
| Clinician | O | O |
| Lab technician | O | O |
| Other | O | O |

Others (please specify) _______________________________________________

In case more than one option suits your profile, please state here the one best describing you ___________________

28. Which of the following educational backgrounds best describes your professional figure?

Please, select one option only.

| Biomedical engineer with a focus on  instrumentation, E-health, and Rehabilitation | O |
| --- | --- |
| Clinical neurophysiologist | O |
| Kinesiologist/Human movement scientist/Sport scientist/Exercise scientist | O |
| Neurologist | O |
| Neurophysiopathology/Biomedical laboratory  technician | O |
| Occupational therapist | O |
| Physical Medicine and Rehabilitation physician, also known as physiatrist | O |
| Physiotherapist | O |
| Speech therapist | O |

Other (please specify) _______________________________________________

29. In which of the following neurological disorders would you expect sEMG to have practical utility in clinical neurorehabilitation?

|  | Not  useful  at all |  | Neutral |  | Very  useful |
| --- | --- | --- | --- | --- | --- |
| Dementia | O | O | O | O | O |
| Epilepsy | O | O | O | O | O |
| Migraine/headache disorders | O | O | O | O | O |
| Multiple sclerosis/demyelinating diseases | O | O | O | O | O |
| Neurodevelopmental disorders | O | O | O | O | O |
| Neuroinfections | O | O | O | O | O |
| Neurological disorders associated with malnutrition | O | O | O | O | O |
| Neuromuscular disorders | O | O | O | O | O |
| Pain associated with neurological disorders | O | O | O | O | O |
| Parkinson’s disease/movement disorders | O | O | O | O | O |
| Peripheral nerve disorders | O | O | O | O | O |
| Spinal cord disorders | O | O | O | O | O |
| Stroke/cerebrovascular diseases | O | O | O | O | O |

Other (please specify) _______________________________________________

Other comments ___________________________________________________

30. In clinical settings, neurorehabilitation is the collection of various expertise to provide the best care and education to patients with injuries or diseases affecting their nervous system. Which of the following best describes your field?

You can select more than one option.

| Help with activities of daily living (ADLs), such as eating, dressing, bathing, toileting, handwriting, cooking, and basic housekeeping | O |
| --- | --- |
| Speech therapy to help with speaking, reading, writing, or swallowing | O |
| Stress, anxiety, and depression management | O |
| Bladder and bowel retraining | O |
| Activities to improve mobility (movement), muscle control, gait (walking), and balance | O |
| Exercise programs to improve movement, prevent or decrease weakness caused by lack  of use, manage spasticity and pain, and maintain range of motion | O |
| Social and behavioral skills retraining | O |
| Nutritional counselling | O |
| Involvement in community support groups | O |
| Activities to improve cognitive impairments, such as problems with concentration,  attention, memory, and poor judgment | O |
| Help with obtaining assistive devices that promote independence | O |
| Patient’s education and counseling | O |
| Pain management | O |
| Stress management and emotional support | O |
| Vocational counseling | O |
| None of the above (I do not work in a clinical setting) | O |

Other (please specify) _____________________________________________
